# Lithotripsy of Calcified Aortic Valve Leaflets by a Novel Ultrasound Transcatheter-Based Device

**DOI:** 10.3389/fcvm.2022.850393

**Published:** 2022-03-25

**Authors:** Giacomo Bernava, Enrico Fermi, Guido Gelpi, Stefano Rizzi, Davide Benettin, Marianna Barbuto, Claudia Romagnoni, Domenico Ventrella, Maria Chiara Palmieri, Marco Agrifoglio, Gianluca Polvani, Maria Laura Bacci, Enrico Pasquino, Maurizio Pesce

**Affiliations:** ^1^Unità di Ingegneria Tissutale Cardiovascolare, Centro Cardiologico Monzino, Istituto di Ricovero e Cura a Carattere Scientifico (IRCCS), Milan, Italy; ^2^AorticLab S.r.l., Bioindustry Park, Colleretto Giacosa, Italy; ^3^ASST Fatebenefratelli Sacco, Milan, Italy; ^4^Università Degli Studi di Bologna, Bologna, Italy; ^5^Università Degli Studi di Milano, Milan, Italy

**Keywords:** calcific aortic valve disease (CAVD), ultrasound, lithotripsy—methods, valve leaflets, valvuloplasty, medical device

## Abstract

The increasing incidence of calcific aortic valve disease necessitates the elaboration of new strategies to retard the progression of the pathology with an innovative solution. While the increasing diffusion of the transcatheter aortic valve replacements (TAVRs) allows a mini-invasive approach to aortic valve substitution as an alternative to conventional surgical replacement (SAVR) in an always larger patient population, TAVR implantation still has contraindications for young patients. In addition, it is liable to undergo calcification with the consequent necessity of re-intervention with conventional valve surgery or repeated implantation in the so-called TAVR-in-TAVR procedure. Inspired by applications for non-cardiac pathologies or for vascular decalcification before stenting (i.e., coronary lithotripsy), in the present study, we show the feasibility of human valve treatment with a mini-invasive device tailored to deliver shockwaves to the calcific leaflets. We provide evidence of efficient calcium deposit ruptures in human calcified leaflets treated *ex vivo* and the safety of the treatment in pigs. The use of this device could be helpful to perform shockwaves valvuloplasty as an option to retard TAVR/SAVR, or as a pretreatment to facilitate prosthesis implantation and minimize the occurrence of paravalvular leak.

## Introduction

Calcific aortic valve disease (CAVD) is one of the most prevalent pathologies in elderly people with a trend to increase worldwide (1–4), along with sex-specific differences (5). Epidemiological studies show that the prevalence of the pathology exponentially increases with age. In addition, the trend is favored by the generalized and continuously growing increase in life expectancy (6) and the constant rate increase in the world population of around 1.05% per year (Worldometer, 2020 global population data; https://www.worldometers.info/world-population/world-population-by-year/). CAVD and other aging-related disorders are therefore anticipated to have an epidemic diffusion in the next decades.

A main characteristic of CAVD is that it has a biphasic trend. During the first phase, named sclerosis, the valve leaflets undergo a slow progression of lipid accumulation and thickening due to the matrix remodeling activity of valve-resident and recruited inflammatory cells. During this phase, the change in valve hemodynamics is minimal. The sclerotic phase of CAVD is relatively slow and is followed by a more rapid stenotic phase, during which the resident cells start releasing small calcified microparticles that accumulate in the matrix (especially at the aortic side of the leaflets), giving rise to large calcific nodules. These nodules distort the leaflet geometry and make leaflets less pliable, preventing complete valve closure and giving rise to regurgitation and valve mechanical insufficiency. This progresses rapidly and can lead to death within 2–3 years after the beginning of the calcification process (7–10). For example, mild aortic valve stenosis (AVS) is defined by a peak velocity between 2.5 and 3 m/s, with a mean trans-valvular pressure gradient of less than 20 mmHg and a valve opening >1.5 cm^2^. By contrast, severe AVS is defined by a peak velocity greater than 4 m/s, a mean gradient of 40 mmHg or more and an aortic valve area less than 1 cm^2^. For several decades, the gold standard treatment of severe aortic stenosis has been surgical aortic valve replacement (SAVR) with mechanical or bio-prosthetic valves. Recently, a new trend has been established by the introduction of transcatheter aortic valve replacements (TAVRs) which has become an optimal solution for patients in the age range of 60–65 to ~80 years (11) or patients who cannot be operated for concomitant risk conditions (12). At the moment, there are no efficient pharmacological therapies that may retard the rapid calcific progression in the stenotic valve or inhibit the transition from the sclerotic to the stenotic phase–a shortcoming for the management of many young patients worldwide.

Inspired by the use of shockwaves to disintegrate kidney stones or gallstones (13, 14), which was introduced in the mid-'80s in medicine, new applications of high-intensity focused ultrasounds have proven to be effective for other treatments for calcific pathologies, such as tendonitis and other orthopedic applications (15). Lithotripsy has also been successfully used in the cardiovascular pathology area as an adjuvant treatment for better implantation of stents in coronary and peripheral calcified arteries (16–18). Similar to lithotripsy, ultrasounds that are properly modulated in intensity, frequency, and waveform can be used to produce fractures and structural changes in calcific aortic valve deposits. For this application, much less energy density is required to limit soft tissue injury. In this study, we describe the first validation of an innovative device that is tailored to disintegrate the calcific deposits in the human aortic valve leaflets. This device has been conceived to be part of a trans-catheter debridement device (TDD) that uses low-intensity ultrasound shockwaves for the calcium ablation in the native aortic or bioprosthetic valves with the aim of restoring the leaflet pliability to, therefore, regain an adequate trans-valvular flow and reduce pressure gradient. We show that treatment with TDD is able to reduce the calcium deposits in human calcified leaflets *ex vivo* without altering the vitality or affecting the structure and gross morphology of the leaflets, and of pericardium as the most represented material normally employed to manufacture bioprosthetic valves. We also show that the employment of the device is feasible in pigs. Based on our results, we propose TDD as an alternative treatment for young patients not eligible for TAVR. It may also be an alternative to the TAVR-in-TAVR procedure when restoring valve performance in patients with preexisting TAVRs affected by *post*-implant calcification.

## Materials and Methods

### Biological Material and Ethics

Human stenotic aortic valves were obtained from a leftover of valve surgery replacement after approval from the Ethical Committee at Centro Cardiologico Monzino for the purpose of the present study. Porcine pericardia were collected from euthanized adult pigs in the experimental piggery facility and the slaughterhouse of the Department of Veterinary Medical Sciences of the University of Bologna. The pig *in vivo* trial was performed at an external facility authorized for the execution of cardiothoracic surgery trials. Animals were treated according to the ethical guidelines approved by the Italian Ministry of Health.

### Description of the TDD Device and of the *ex vivo/in vivo* Treatment Protocol

**Figure 1:**
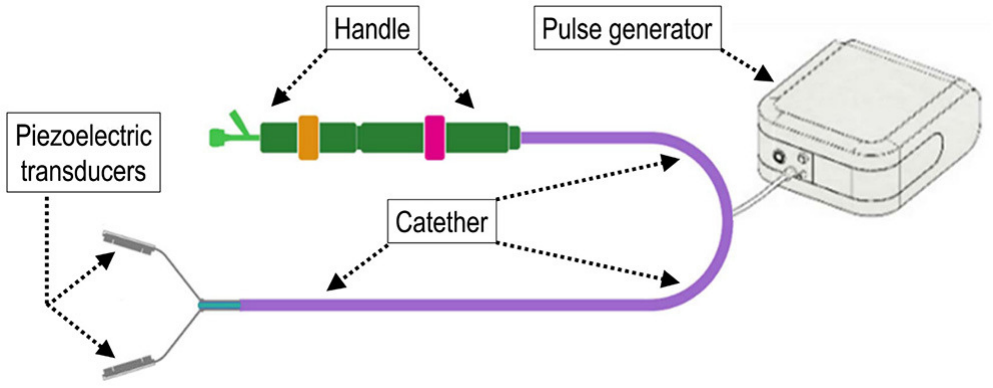
Scheme illustrating the main components of the trans-catheter debridement device (TDD) in the configuration as it was employed for the *in vivo* trial. The device consists of two piezoelectric transducers that can be juxtaposed to deliver shockwaves on both sides of the region of treatment. The impulse-transducing cables are inserted into an intravascular catheter and operated by a handle at the other extremity to deploy and de-sheath the device.

The TDD used in the present study was an evolution of the device we preliminarily tested (19). The theoretical considerations underlying the use of shockwaves is provided for interested readers in the supplementary information. Briefly, the TDD comprised a pulse wave generator that was designed to provide two narrow impulsive electrical signals ranging from 50 to 75V. For the present study, one electrical signal was emitted pulses at 100 kHz and the other emitted at 3MHz to the ablation unit in an alternating pattern, with a time interval of 6 s. The combination of these different frequencies improved the disruptive effects of calcium deposits in the aortic valve cusps, avoiding thermal injury and the breaking of the transducers. The ablation unit was based on two mechanically bound piezoceramic transducers produced by PI Ceramics that had dimensions of 2.7 mm × 8 mm ×.7 mm. Each transducer was electrically connected to a Kapton flexible printed circuit and housed in metal support. The metal support was engineered to create a backing effect on the ultrasounds emitted by the transducer as the waves are conveyed in the direction of treatment. To obtain this effect, the thickness of the wall where the transducer was anchored is $\frac{3}{4}\lambda$. The transducer must be electrically isolated to work in a biological environment. The insulating material was a variant of parylene. It is deposited with a thickness of $\frac{\lambda }{4}$ that facilitates the passage of ultrasonic waves as the effects of refraction and reflection are limited. The debridement device used for the execution of the tests that is reported in this article consisted of a clamp whose faces are constituted by two piezoceramic transducers, but the final version of the device also included an artificial temporary valve with a Nitinol support structure intended to be positioned within the native valve to keep it open during the positioning of the transducers. Figure 1 illustrates the main constructive elements of the TDD and its experimental setup for the removal of calcium deposits in the soft valve tissue. The calcific leaflets were continuously treated for 30 min at alternating frequencies of 3 MHz and 100 KHz, with a switch between the two frequencies every 6 s. The combination of two-frequency ultrasound fields, as indicated in the literature (REF), increases the cavitation effects compared to the use of a single frequency. Hence, it is likely to preserve the integrity of the transducer and reduce the heat emitted.

### TDD Treatment of Porcine Pericardium

Immediately after gentle isolation of the parietal pericardial membrane, the tissue was immersed in a solution of Phosphate-buffered saline (PBS)-containing antibiotics (penicillin/streptomycin) and stored or shipped at 4°C until the beginning of the experiments. Before the treatment with shockwaves, the remaining fat tissue was removed from the pericardial flap under sterile conditions. Subsequently, it was divided into stripes with a sterile knife and further cut into three samples/stripes. TDD treatment was localized at the center of the samples after marking with a surgical pen for discriminating the treated vs. the untreated portions in subsequent analyses.

### Treatment of Human Valves With TDD

Candidate valves for TDD testing were selected on the basis of the degree of calcification as detected by echocardiographic characteristics before the valve substitution intervention. In general, valves with low or moderate stenosis levels were selected due to the inability of our experimental TDD to ablate nodules with a volume smaller than 100 mm^3^. The system was mounted with the two piezoelectric transducers clamping the portion of the tissue to be treated before connecting to the system amplifier (Figure 1).

### *In vivo* Pig Model

The experimental treatment was carried out in two adult pigs in a fully equipped operating room with a portable angiographic C-arm. Under general anesthesia, the animals were fully heparinized, and the femoral artery was exposed to perform the treatment with TDD using a minimally invasive procedure. The animals were kept anesthetized for the whole duration of the treatments and were immediately euthanized after the procedure to recover the heart and dissect the treated valves. The mean arterial pressure was stable during the entire procedure (mean arterial pressure of 80 mmHg with heart rate of ~130 beats per min). No moderate to severe aortic regurgitation was ever detected by echocardiographic monitoring during and after TDD treatment. No pacing or rapid pacing was needed for the treatment.

### Histological Characterization

Human and pig valve samples were fixed in 4% paraformaldehyde (4°C overnight), dehydrated in alcoholic scale, and embedded in paraffin. Histological sections (5 μm in thickness) were cut, dewaxed, and stained. Von Kossa staining was performed to evaluate calcium deposits in untreated vs. treated portions of the specimens. Conventional staining (hematoxylin/eosin, Masson's trichrome) was performed to observe in greater detail the structure of the tissue. Images of the tissue sections were acquired using an AxioPlane optical microscope (Carl Zeiss) and analyzed with ZenBlue software.

## Results and Discussion

### Validation of TDD *in vitro* and *ex vivo*

To preliminarily test the efficiency of calcium deposits debridement in human aortic valves, experiments were performed on explanted and formaldehyde-fixed aortic valve leaflets. Under these conditions, we obtained an average volume reduction of 13.87% with a single piezo and a supply voltage of 55V, which allowed to obtain a peak of acoustic pressure of .2Mpa. The emitted energy is 150 mj/mm^2^ in the center of the transducer and 80 mj/mm^2^ at the edges (the shape of the emitted field is pyramidal). Volume reduction was calculated from the post-treatment vs. pre-treatment leaflet by CT scan (Supplementary Figure S1).

**Figure 2:**
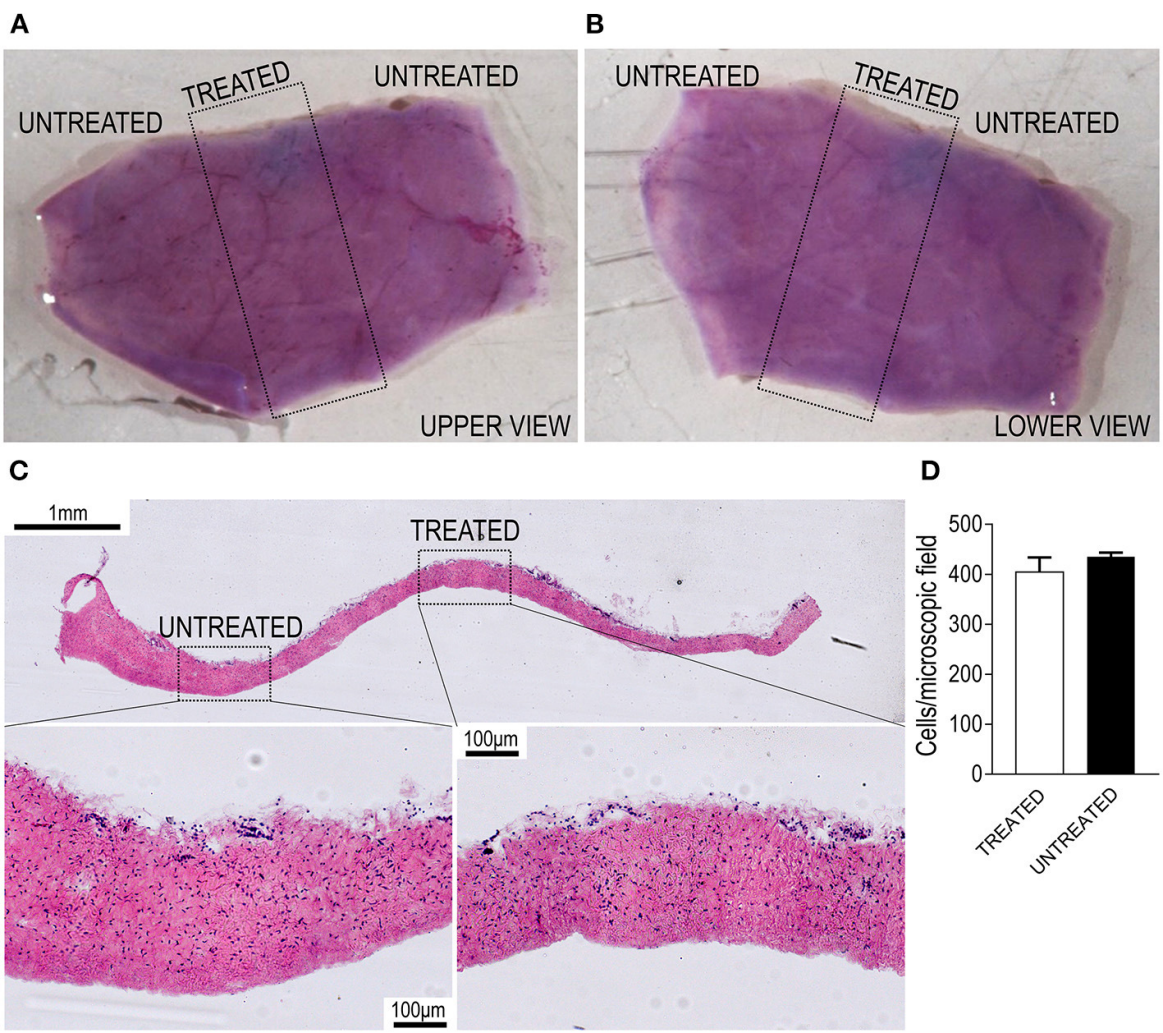
Validation of the TDD on living porcine pericardium. **(A,B)** Show the two sides of a pericardial patch stained with 3-(4.5-dimethylthiazol-2-yl)2,5-diphenyltetrazolium bromide (MTT; purple color). The region encircled by the dotted rectangle indicates the part that was exposed to shockwaves and does not exhibit any staining reduction, suggesting maintenance of an excellent vitality of the tissue. **(C)** Shows the transversal section of another section of living pericardium treated with the TDD. It is evident from the high magnifications in the lower part of the panels that cells in the treated regions are in the same amount as in the untreated portions. Quantifications of nuclei are present in **D**, showing no difference in the nuclei counting in treated vs. untreated portions. Bar graph displays the mean and the SE of data (*n* = 3 independently treated pericardial samples; *p* > 0.05 by paired Student's *t*-test).

Since application of shockwaves to tissues can determine mechanical damages to cells, we were prompted to assess the effects on cellular survival and tissue ruptures using our experimental setup. This was done by measuring the vitality of living pericardial membranes from pigs, a tissue that we already employed for engineering valve tissues after recellularization (20) and is the golden standard material for manufacturing valve replacements. As shown in Figures 2A,B, the samples, stained with 3-(4.5-dimethylthiazol-2-yl)2,5-diphenyltetrazolium bromide (MTT) and treated with the device, exhibited no differences in the overall vitality of the treated vs. the non-treated areas on both sides of the tissue. The absence of large ruptures and the presence of cells with normal morphology in transversal sections of the treated and untreated areas showed that the type and intensity of the shockwaves delivered by the TDD did not affect, at least macroscopically, the integrity of the tissue (Figure 2C). Confirming the macroscopic observations by MTT staining, a normal amount of cells in the treated vs. the non-treated areas were counted in high magnifications of transversal tissue sections of the pericardial material (Figures 2C,D). This *bona fide* consolidates the lack of important structural deterioration of a valve-resembling living tissue treated with the device and warrants the safety of the procedure for employment *in vivo*.

**Figure 3:**
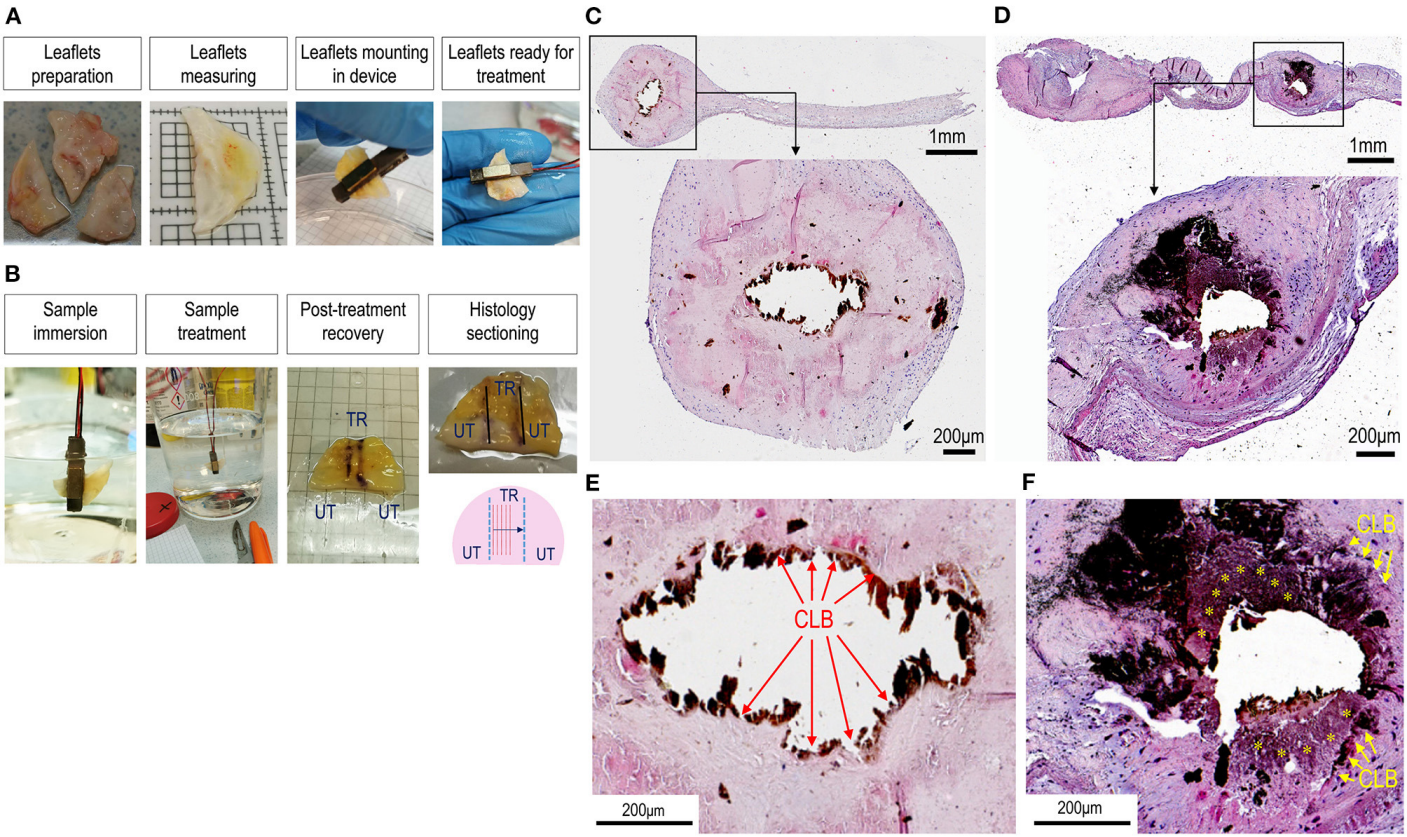
Treatment of calcific aortic valve disease (CAVD) leaflets by TDD. **(A,B)** Show the sequence of the human CAVD leaflets mounting in the debridement device. After preparation, leaflets were individually placed among the two piezoelectric transducers and immersed into a phosphate-buffered saline (PBS) solution for the treatment. After treatment, the orientation of the treated zones (TR) was marked with a surgical pen to distinguish them from the untreated portions (UT). These marks were taken as a reference for histological sectioning that occurred in a transversal orientation as indicated in the lower right panel in **(B)**. **(C,D)** And the high magnifications of the histological regions represented therein **(E,F)** show the difference in the aspect of the calcifications in control **(C,E)** vs. the treated calcific leaflets **(D,F)**. It is remarkable that in control tissues there was a net boundary between the calcific lesions (removed by sectioning; CLB) and the surrounding sclerotic tissue (red arrows in **E**). By contrast in treated tissues, the boundaries were less sharp (yellow arrows) and granular-like deposits (yellow asterisks) appeared around the lesions, suggesting a partial debridement of the calcium. See also Supplementary Figure S1 for another clear example of calcium debridement in a calcific human valve leaflet treated with TDD. Sections in **C–F** stained with von Kossa staining solution.

To validate the double-piezo device for fragmentation and/or disruption of the calcium deposits in living calcified valves, we employed the TDD to treat calcified areas of human stenotic valves leaflets that were freshly explanted from patients with CAVD who were undergoing surgical valve replacement (Figure 3A). Treatments were performed using a 3 MHz ultrasound field combined with a field at a low frequency of 100 kHz, which, according to preliminary tests, were found to ensure the most efficient removal of the nodules of intermediate or low size (up to 100 mm^3^). To this aim, we selected areas of the leaflets that were not characterized by the largest calcific deposits or affected by sclerosis, where, instead, calcium deposition is not involved (21). Leaflets were treated in areas juxtaposed to the visible calcifications with an orientation, as indicated in Figure 3B. From the morphological observation of the leaflets after treatment, the tissues, in no case, showed signs of burns or obvious damages. Histology staining was performed after transversally cutting the treated areas to analyze possible microscopic ruptures to the extracellular matrix components (e.g., collagen and elastic fibers) and variations in the dimensions of the calcium nodules. Figures 3C–F show representative low or high magnifications of calcifications in control (Figures 3C,E) and treated (Figures 3C,F, Supplementary Figure S2) leaflet sections stained with von Kossa. In treated leaflets, it was evident that a partial fragmentation or disruption of the calcium deposits appeared less compact than in untreated samples, as shown by the reduction of the area covered by the compact bone, and the presence of areas where the calcium appeared pulverized. The elimination of calcium deposits was also macroscopically observed by treating a calcified human valve in a cadaveric heart (Supplementary Figure S2). Together, these data macroscopically and microscopically demonstrate the removal of calcium nodules from the calcific leaflets by TDD.

### Safety of TDD Employment *in vivo*

**Figure 4:**
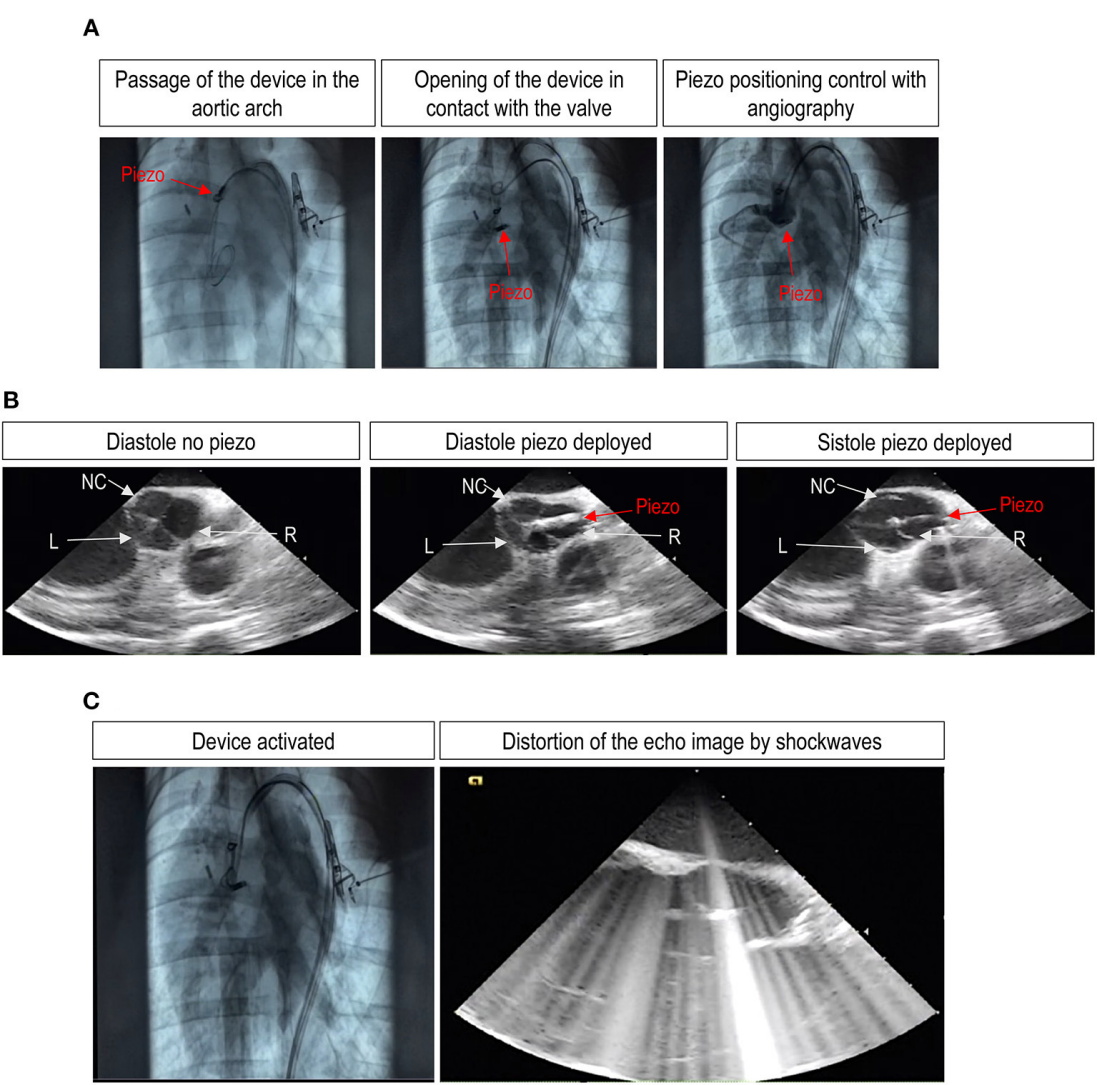
Angiographic and echocardiographic assessment of successful TDD deployment and leaflet treatment with a trans-femoral access *in vivo*. **(A)** Shows the deployment of the device through the femoral artery from an initial position at the level of the ascending aorta and down to the positioning of the piezo in the Valsalva sinus of the right aortic valve leaflet. **(B)** Shows three images of an echocardiographic sequence showing the motion of the valve leaflets in the presence of the piezo deployed at the level of the right leaflet. The pictures of the sequence show, respectively, the correct closure of the three leaflets before piezo deployment (left picture), the closure of the valve after piezo deployment (center picture), and the opening of the left (L) and the non-coronary (NC) leaflets or the blockade of the right (R) leaflet by the deployed piezo at systole (right picture). The complete sequence is provided as Supplementary Video 1. **(C)** Shows, finally, static images of the angiography and the echocardiographic sequences during activation of the piezo. It is evident on the right the interference of shockwaves with the ultrasounds, making it impossible to distinguish the structure of the valve.

A pigtail catheter, under fluoroscopic guidance, was advanced through a 6 Fr arterial introducer and over a 0.035″ standard wire until the right aortic valve sinus of Valsalva to mark aortic valve plane and leaflet. The TDD, integrated in a transfemoral 21 Fr delivery system, was advanced over a 0.035″ stiff wire through the thoracic aorta and aortic arch to the aortic root (Figure 4A, left center, Supplementary Video 1). An angiography was performed to highlight the correspondence between the position of the device and the aortic valve plane (Figure 4A, right, Supplementary Video 1). After opening the device, under intravascular ultrasound control (Figure 4B), the active element was correctly positioned by laying on the aortic side of the leaflet. The device only kept in closed position at which the leaflet was lying on, allowing the two other leaflets to normally open and close for the whole duration of the treatment (Figure 4B, center and right) and after retraction of the TDD from the valve (Figure 4C and Supplementary Video 1). It was interesting to note that during the activation of the piezo, the TDD ablator interfered with the echo signal. While this suggests an efficient *in vivo* functioning of the device, it also shows that echocardiographic assessment of leaflet motion during ultrasound treatment is not possible, thus leaving to angiographic monitoring the elective way to control the positioning of the piezo during the procedure. Every 10 min, the treatment was interrupted to check the status of the electrical insulation of the device. The check was carried out by pinching the electrical cables connected to the generator, and the piezoelectric capacity was measured with a tester.

**Figure 5:**
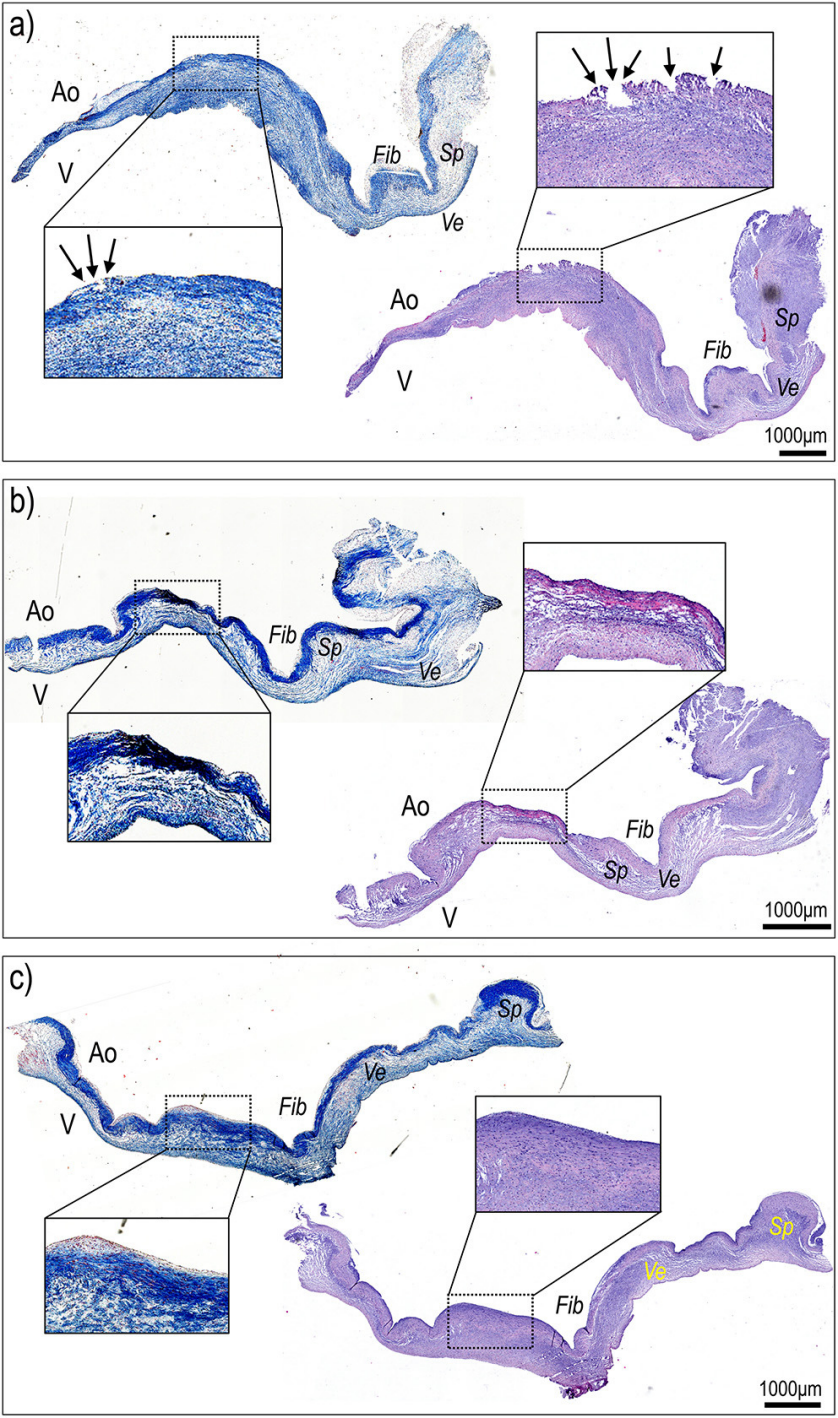
Histological sectioning of the right **(a)**, non-coronaric **(b)**, and left **(c)** leaflets of the aortic valve treated with TDD in Figure 4 from commissure to commissure and along a radial direction stained with hematoxylin/eosin and Masson's staining solutions. The right leaflet, treated with the TDD **(a)**, showed evident signs of superficial damage (arrows in magnification inset) that were contained within the superficial portion of the fibrosa (Fib) identifiable on the aortic (Ao) side of the valve due to its intense blue staining. In no case the ventricular portion (V) or the ventricularis (Ve) or the spongiosa (Sp) layers exhibited signs of tissue damage.

After the end of the experiments, the valves were explanted and processed for histological sectioning. Given that the entire valves were available, the three leaflets were individually dissected from the Valsalva sinuses and transversally sectioned in a circumferential direction from commissure to commissure to screen for potential tissue damage. Figures 5a–c show the picture of the right, left, and non-coronary leaflets, respectively, of one of the valves treated by the TDD *in vivo*. To identify possible tissue damages, staining with Masson's and Hematoxylin/Eosin (H&E) solutions were performed. The magnifications in each of the panels indicate areas with possible tissue damage due to the treatment. As expected, evident signs of mild/moderate ruptures on the aortic side of the right leaflet (Figure 5a), the one that received the ultrasounds according to echocardiography data. These ruptures, however, were found only on the most external surface of the tissue and did not affect the collagenous structure of the *fibrosa* layer, suggesting that the shockwaves were very focused on the area of the tissue localized immediately below the contact point of the piezoelectric transducer to the aortic side of the leaflet. This finding, in keeping with the data obtained in animal pericardium and the human calcific leaflets, shows a localized effect of the TDD that is tailored to fracture the calcific lesions without involving major tissue ruptures.

## Conclusions and Limitations

In the present study, we provide the first proof of concept demonstration of a new device specifically tailored to operate with a high level of safety and efficiency. It is a partial debridement of the calcific deposits affecting the aortic valve motion and hydrodynamic performance. Although innovative, this approach is not the first to be employed to this aim. In fact, in recent literature, there are emerging descriptions of lithotripsy application as a preventive strategy to improve the ellipticity index and limit the paravalvular leak of transcatheter aortic valve implantations (TAVIs) (22, 23), to reduce percutaneously the extent of cardiac valves calcifications (24), or as a pretreatment for valvuloplasty (25, 26). On the other hand, the devices employed so far to deliver shockwaves to the valves—expandable lithotripsy balloons tailored for intravascular calcific lesions debridement—are not specifically designed for the scope of shockwave-based valvuloplasty and, therefore, do not have the design specifications to perform debridement of the calcific lesions with the necessary precision and accuracy. The TDD presented in our investigation has been conceived from the beginning as a specific device for treatment of the valve leaflets with calcifications. Compared with the existing lithotripsy balloons, TDD has, for example, the possibility to be placed precisely on the portions of the leaflets to be treated under angiographic and echocardiographic guidance. In addition, although the system employed in our *in vivo* trial consisted of only one piezoelectric transducer, by virtue of the double piezo design that could be implemented in a clinical version, the TDD offers the advantage to treat both sides of the leaflets and operate with shockwaves with the desired power and frequency to optimize calcium debridement, thereby minimizing tissue damages. Although the system has been designed to reduce the size of the calcium deposits in valves with calcific disease and positively impact on the valve hemodynamics with the recovery of leaflet pliability, another appealing opportunity to employ our system is also to remove calcium deposits from calcified biological prostheses in preparation of TAVR-in-TAVR procedures, to restore leaflets pliability and valve performance in patients with calcified TAVRs, and to reduce the content of the calcium in the valve annulus to reduce paravalvular leak in conventional SAVR with mechanical or bioprosthetic implants. In this regard, the increase in energy delivery (up to 150 V and the presence of a double piezo on both sizes of the leaflets) planned in the next version of the *in vivo* device will help to improve the efficiency of calcium deposits debridement. Accordingly, suitable mechanical tests in a pulse duplicator system will be necessary on treated valves to confirm the variations in hemodynamics after treatment with TDD in its full power configuration.

A possible limitation in the transfer of TDD to the clinics may result from potential pathologic effects of the shockwaves resulting from induction of cellular apoptosis or local or systemic inflammation (27–30) that could lead to rapid tissue re-calcification. In the present study, our primary concern was to demonstrate that delivery of the shockwaves does not determine macroscopic ruptures in the leaflets and the pericardium. Hence, it does not affect the survival of cells inside the treated tissues and performs efficient calcium debridement. Future safety assessment of the TDD will have to be performed *in vivo*, ideally using animal models, i.e., sheep (31), where the possible pathologic evolution of the treated leaflets will be monitored with state-of-the-art systems.

Other important risks that should be considered for clinical implementation of the device are the potential embolization of calcific debris and the thrombogenic effects of possible valve leaflets denudation. Although in the version of the TDD employed in the present study, this component was not present. Our device has been designed to be used with deployment of a distal filter to avoid unwanted debris embolization. To limit the risk due to endothelial denudation and thrombogenicity, tests will finally be conducted with the administration of anticoagulation therapy in animals treated with TDD to monitor the dynamics of reendothelialization of the portions of treated leaflets. In summary, due to the possibility to modulate the frequency and the power of the shockwaves, our new device offers the possibility to promote, at least potentially, *in vivo* valve tissue repair as it has been demonstrated, for example, for the kidney (32).

## Data Availability Statement

The raw data supporting the conclusions of this article will be made available by the authors, without undue reservation.

## Ethics Statement

The studies involving human participants were reviewed and approved by Ethical Committee - Centro Cardiologico Monzino, IRCCS. The patients/participants provided their written informed consent to participate in this study. The animal study was reviewed and approved by Ministero della Salute.

## Author Contributions

EF, DB, MCP, and EP: concieved the device. GB, CR, GG, SR, and MB: performed experiments. DV, MLB, MA, and GP: provided samples. GB, EF, EP, GG, and MP: revised data and analyzed results. MP: wrote the paper.

## Funding

GB, GG, SR, MB, CR, MA, GP, and MP are supported by institutional funding (Ricerca Corrente, Ricerca 5 per mille – Ministero della Salute, Italy). DV and MLB are supported by the local funding program RFO-UNIBO.

## Conflict of Interest

EF, DB, MCP, and EP are collaborators of the equity-financed company AorticLab S.r.l. AorticLab S.r.l. is an equity-financed Company for development of the TDD as a novel minimally invasive calcium debridement procedure in human pathologic valves. GG and CR were employed by ASST Fatebenefratelli Sacco. The remaining authors declare that the research was conducted in the absence of any commercial or financial relationships that could be construed as a potential conflict of interest.

## Publisher's Note

All claims expressed in this article are solely those of the authors and do not necessarily represent those of their affiliated organizations, or those of the publisher, the editors and the reviewers. Any product that may be evaluated in this article, or claim that may be made by its manufacturer, is not guaranteed or endorsed by the publisher.
