# Supplementary material for: Lithotripsy of Calcified Aortic Valve Leaflets by a Novel Ultrasound Transcatheter-Based Device
Source: Front Cardiovasc Med. 2022 Mar 25;9:850393. doi: 10.3389/fcvm.2022.850393 (PMC8990875; doi:10.3389/fcvm.2022.850393)
Supplement: Supplementary file 2 [file Data_Sheet_1.pdf]

Lithotripsy of calcified aortic valve leaflets by a novel ultrasound transcatheter-based device

Giacomo Bernava[1,*], Enrico Fermi[2,*], Guido Gelpi[3], Stefano Rizzi[1], Davide Benettin[2], Marianna Barbuto[1], Claudia Romagnoni[3], Domenico Ventrella[4], Maria Chiara Palmieri[2], Marco Agrifoglio[5], Gianluca Polvani[5], Maria Laura Bacci[4], Enrico Pasquino[2] and Maurizio Pesce[1,§]

Supplementary material

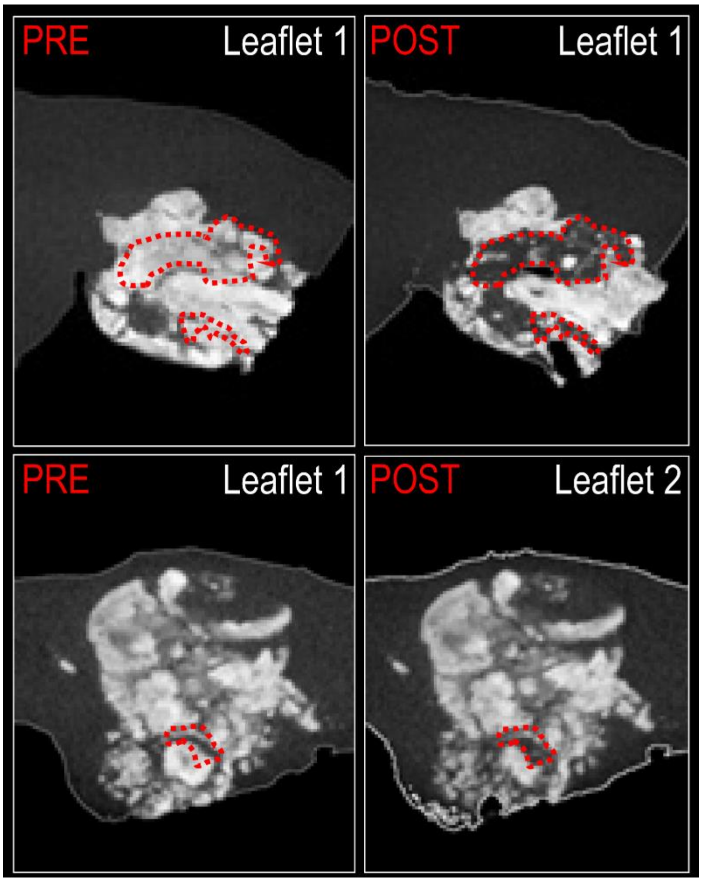

Figure S1. CT scan of calcified leaflets before (PRE) and after (POST) treatment with TDD. The two leaflets exhibited a reduction of the calcium deposits in the areas encircled in red.

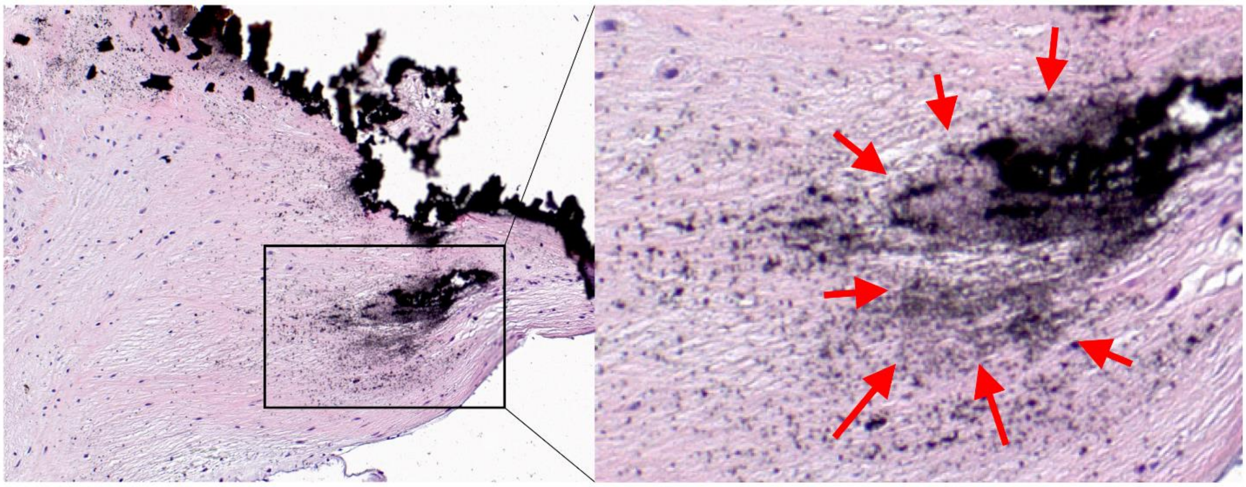

Figure S2. Particular of another calcific lesion of human CAVD leaflets treated with TDD, exhibiting evident signs of calcium debridement (red arrows).

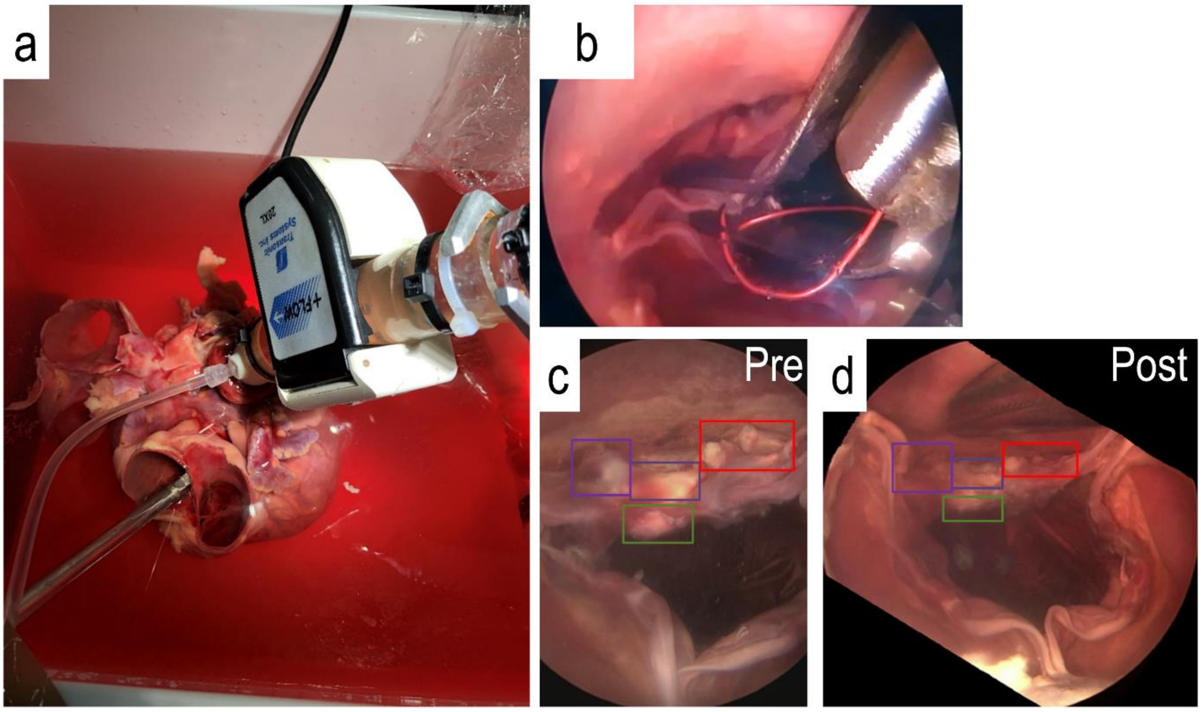

Figure S3. Treatment of a calcific valve in a cadaveric heart was performed to confirm the reduction in the extent of calcification by TDD in a whole human heart condition. As shown by the reduction of the white deposits in panel **d** versus panel **c** (see rectangles in color), TDD appeared to remove a significant portion of the calcific lesions in the aortic valve.

Supplementary materials and methods

Shock waves and their biophysical effects.

In literature the mechanism of action of ultrasound for disrupting calcific concretions is widely described (1, 2)

The ultrasound field can be produced in three different ways (3):

- electro-hydraulic generator: two electrodes, crossed by current, overheat the water in which they are immersed. This causes evaporation and consequently the rise in pressure that generates the shock wave.
- electromagnetic generator: a coil wrapped in a metal membrane generates a magnetic field when the current passes which causes the membrane to expand, thus causing the formation of shock waves
- piezoelectric generator: the system uses piezoelectric crystals, or transducers, immersed in water which, undergoing contractions and expansions of their volume, produce very small pressure waves in the water

For the design of TDD piezoelectric transducers are used to create low-intensity energy waves. The piezoelectric effect is the skill of a material to generate mechanical stress when subjected to a potential difference. The effect is due to the distortions suffered by the crystal lattice of the material. The transducer is like a RLC circuit: when injecting a current that has a frequency equal to the resonant frequency of the transducer, the impedance tends to zero, increasing the mechanical effects of vibration by emitting ultrasound (4).

The shock wave is characterized by a positive pressure pulse of short duration and a negative pressure pulse. The pressure curve that describes the shock wave is characterized by an ascending phase in which the rise time (Tr) can vary from a few nanoseconds (ns) to a few microseconds (μs) and represents the time that the pressure takes to rise from 10% to 90% of its maximum value (Pmax). The wave trend in the descending phase of the curve is instead slower and more irregular, before assuming a negative value.

More in detail the shockwaves, constituted at least by two components namely direct compression and negative tension, act on the calcific deposits with a combination of multiple effects (5):

- spallation that occurs when the shockwave crosses the calcification and is reflected on the back wall. The reflection of the impulse results in a mechanical tension that is more effective than the compression force (incident impulse);
- shear forces that result from a combination of compressive and transverse waves. This effect can be very effective on the calcific deposits since the stratified and fragile conformation of calcium concretions has low resistance to transverse shear forces;
- reflection of refracted waves that is generated from the reflection of the pressure waves during the crossing of the concretion;
- cavitation of the bubbles that consists in the nucleation of bubbles near the calcific deposits in the blood and subsequent dynamics involving growth, oscillation and collapse with the formation of microjets;

- fatigue that can cause the breakage of the calcific deposits when subjected to mechanical stimuli. This occurs usually where there are imperfections in which the effects of shockwaves are concentrated.
- superfocusing that is created by the geometry of the calcification as a combination of reflections and refractions of the waves that are focused within it.

As already reported by Fermi E. et al. doi:10.26717/bjstr.2021.33.005429, cavitation is a fundamental phenomenon for the breakdown of calcium that forms inside the leaflets. The transcatheter debridement device is, in fact, based on histotripsy, an experimental cavitation-based therapy in which ultrasounds "break" the fluid they pass through, forming bubbles of dissolved gas. The gas bubbles, subjected to the ultrasonic field, undergo compression and decompression forces, transforming the bubble into an oscillating system that can explode. The explosion of the bubble causes mechanical erosion due to the concentrated release of energy. The bubble oscillates elastically according to the gas inside it and according to the liquid that surrounds it: this means that the bubble has its own oscillation frequency. The variation in the radius of the bubble is inversely proportional to the frequency described above.
The following equation can describe the resonant frequency of the bubble in fluid:

$$f = \frac{1}{2\pi R\sqrt{\rho}}\sqrt{3k(\rho_0 - \rho_g + \frac{2\sigma}{R}) - \frac{2\sigma}{R} - \frac{4\mu^2}{\rho R^2}} (6)$$

where p_0 is the environmental pressure, k the polytropic index, R the bubble radius, p_g the gas pressure in the bubble, ρ the density of the surrounding medium, σ the surface tension and μ the viscosity.
This relationship can be simplified in this way:

$$R \approx \frac{3}{f}$$

Cavitation threshold is closely related to the initial bubble radius. Cavitation is induced at frequencies between kHz and MHz. The minimum frequency of a shock wave source, which induces cavitation phenomena, is given by the definition of the mechanical index or MI:

$$MI = \frac{P_{neg}\,(MPa)}{\sqrt{f}} (7)$$

At the peak of negative pressure, Pneg corresponds to the maximum rarefraction of the acoustic wave.
Cavitation occurs when MI>$0.7\sqrt{f}$.
When the frequency of the ultrasound field comes close to the proper bubble frequency, resonant phenomena occur: the bubble dilates during the negative phase of the pressure wave and collapses very quickly and violently upon the arrival of the positive pressure.
The combination of multiple frequencies affects the formation of bubbles leading to the formation of a larger number of bubbles with different rays: the high frequency generates small bubbles locally, while low-frequency stimulation generates large bubbles while the combination of the two frequencies increases the generation rate of cavitation bubbles. The lower frequency must be approximately thirty times lower than the high frequency. This effect forms a number of bubbles that are up to five times larger compared to the use of a single frequency. Furthermore, the non-linear effects together with the combination of the two frequencies reduce the threshold to generate the cavitation effects. Another important element

for the cavitation is represented by the Bjerknes forces which intensify the fragmentation of the bubbles due to the interaction of the bubbles themselves (8).
The following integral provides the estimate of the acoustic pressure given by the sum of the contributions of a source to r^1 towards point r:

$$\hat{p}(x,y,z) = \frac{i\rho ck}{2\pi}\int_S \frac{ue^{-ik(r-r^1)}}{(r-r^1)} ds \ (9)$$

where ρ is the density of the tissue, c the speed of sound, k the wavenumber, u is the complex surface speed.
For two excitation frequencies, the absolute value of p becomes:

$$p_{mixed}(x,y,z) = \left|\hat{p}_{f1(x,y,z)} + \hat{p}_{f2/(x,y,z)}\right| \quad (9)$$

where f1 and f2 represent two different frequencies of stimulation.
This demonstrates that combining at least 2 different frequencies an amplification of the effect of the ultrasound field is obtained.
The combination of two frequencies accelerates bubble collapse: cavitation bubbles become more unstable and easier to collapse under the pressure of dual-frequency ultrasound. With a dual-frequency ultrasound field, the pressure inside the bubble is higher than that obtained with a single frequency. For this reason, the dual-frequency field intensifies the cavitation effect.

Supplementary references

1. D. J. Kereiakes, R. Virmani, J. Y. Hokama, U. Illindala, C. Mena-Hurtado, A. Holden, J. M. Hill, S. P. Lyden and Z. A. Ali: Principles of Intravascular Lithotripsy for Calcific Plaque Modification. *JACC Cardiovasc Interv*, 14(12), 1275-1292 (2021) doi:10.1016/j.jcin.2021.03.036
2. E. Fermi, D. Benettin, G. Bernava, M. Pesce and E. Pasquino: Trans-Catheter Double-Frequency Ultrasound Ablator for The Treatment of Aortic Valve Leaflets Calcification. *Biomed J Sci & Tech Res*, 33(4), 25952-57 (2021) doi:10.26717/BJSTR.2021.33.005429
3. T. Leong, M. Ashokkumar and S. Kentish: The fundamentals of power ultrasound - A review. *Acoustics Australia*, 39 (2011)
4. H. Jaffe: Piezoelectric Ceramics. *Journal of the American Ceramic Society*, 41(11), 494-498 (1958) doi:10.1111/j.1151-2916.1958.tb12903.x
5. J. A. Ogden, A. Toth-Kischkat and R. Schultheiss: Principles of shock wave therapy. *Clin Orthop Relat Res*(387), 8-17 (2001) doi:10.1097/00003086-200106000-00003
6. H. Dong, X. Zou and S. Qian: Simulation Study on the Influence of Multifrequency Ultrasound on Transient Cavitation Threshold in Different Media. *Applied Sciences*, 10(14), 4778 (2020)
7. T. G. Leighton: 4 - The Forced Bubble. In: *The Acoustic Bubble*. Ed T. G. Leighton. Academic Press, (1994) doi:https://doi.org/10.1016/B978-0-12-441920-9.50009-2
8. G. Iernetti, P. Ciuti, N. V. Dezhkunov, M. Reali, A. Francescutto and G. K. Johri: Enhancement of high-frequency acoustic cavitation effects by a low-frequency stimulation. *Ultrasonics Sonochemistry*, 4(3), 263-268 (1997) doi:10.1016/s1350-4177(97)00034-5
9. H. L. Liu and C. M. Hsieh: Single-transducer dual-frequency ultrasound generation to enhance acoustic cavitation. *Ultrason Sonochem*, 16(3), 431-8 (2009) doi:10.1016/j.ultsonch.2008.08.009
